# Mapping ecological targets and outcome evaluations in arts-based interventions for women survivors of domestic violence: a scoping review

**DOI:** 10.3389/fpubh.2026.1897444

**Published:** 2026-07-17

**Authors:** Xingcen Liu, Kamal Sabran

**Affiliations:** School of the Arts, Universiti Sains Malaysia, Penang, Malaysia

**Keywords:** arts-based interventions, domestic violence, outcome evaluation, scoping review, social ecological model

## Abstract

Domestic violence remains a major public health challenge with profound consequences for women survivors. Arts-based interventions are increasingly incorporated into community services as culturally responsive recovery approaches for marginalized populations; however, it remains unexamined whether quantitative evaluation frameworks adequately capture their broad intended outcomes. This scoping review mapped the literature on arts-based interventions for survivors, examining the ecological alignment—or potential evaluative tension—between stated objectives and quantitative evaluation metrics. Conducted following established scoping review methodology and reporting guidelines, systematic searches of Web of Science, Scopus, PubMed, and PsycINFO identified 12 eligible empirical studies. Data were charted using the Social Ecological Model across individual, interpersonal, and community levels. Findings revealed interventions were predominantly community-integrated, with objectives extending beyond psychological symptom reduction to encompass peer connection, cultural belonging, and collective healing. While individual-level recovery was evaluated using congruent psychological measures, broader interpersonal and community-oriented goals were less consistently represented. This revealed an evaluative tension, wherein relational and community outcomes were only partially reflected in formal quantitative assessments. Although arts-based interventions operate across multiple ecological levels, current evaluations remain disproportionately focused on individual psychological outcomes. Future research and policy initiatives may benefit from developing ecologically responsive measurement approaches that capture relational and community-level outcomes alongside clinical measures, thereby providing rigorous evidence to inform the funding, implementation, and evaluation of public health programs for women survivors of domestic violence.

## 1 Introduction

Domestic violence (DV) and intimate partner violence (IPV) represent pervasive and severe public health issues that exert profound, life-course impacts on the physical, psychological, and social well-being of survivors (1). While DV broadly encompasses abuse within various domestic settings, IPV specifically denotes physical, sexual, or psychological harm inflicted by a current or former romantic partner (2). Given their inextricably linked and overlapping nature in public health discourse, this review encompasses both intersecting phenomena. Beyond immediate physical harm and documented clinical trauma, such as post-traumatic stress disorder (PTSD) and depression (3, 4), DV and IPV further reinforce gender and health inequalities. The resultant long-term trauma frequently erodes female survivors’ sense of self-worth and impairs their capacity for social connection, leading to emotional and structural isolation (5, 6). Consequently, public health interventions for female survivors of violence should extend beyond individual symptom management. Contemporary trauma-informed frameworks increasingly emphasize that effective support requires not only alleviating psychological symptoms but also addressing underlying structural stressors while rebuilding relational safety and personal autonomy for survivors (7, 8).

While coordinated systems across health, legal, and social services remain the foundation for supporting DV and IPV survivors (9), arts-based interventions are increasingly integrated into public health and community support practices as innovative components of these cross-sector responses (10). Comprehensive syntheses, including reports from the WHO, indicate that arts practices, as nonverbal forms of support, may help promote psychological health and community well-being (11). Particularly for marginalized populations facing complex structural barriers, community-integrated arts programs can provide a more culturally responsive pathway to support. By utilizing painting, video, music, and other creative modalities, these interventions offer survivors relatively safe spaces for trauma expression and social participation, effectively bypassing the limitations of traditional verbal expression and cognitive narratives in trauma communication (12). For survivors of DV and IPV, trauma is frequently compounded by shame, emotional suppression, and difficulties in verbal disclosure; therefore, expressive practices grounded in nonverbal and somatic experiences hold particular significance within trauma-informed support systems (13).

However, despite the growing application of participatory arts practices in community and nongovernmental organization (NGO) settings, translating these into sustainable, evidence-based public health policy and practice remains challenged by significant methodological limitations in outcome measurement (14). Current evaluation approaches in arts-based health interventions frequently utilize standardized psychological measures to assess changes in individual symptoms such as anxiety, depression, and trauma-related distress. However, previous methodological literature has noted that these measures may not fully capture the broader social, relational, and community-level dimensions of arts-based interventions, particularly within complex community settings (14, 15). While these quantitative metrics are valuable for capturing psychological fluctuations, they may not fully reflect the broader social value of arts interventions regarding social relationships, group support, and community participation. However, it remains systematically unexamined whether existing quantitative evaluation frameworks effectively capture the broader social, relational, and community-level dimensions intended by these arts-based interventions. Exploring this potential ecological alignment—or mismatch—is critical for determining the extent to which broader relational and community-level outcomes are captured within existing evaluation frameworks.

Given the high heterogeneity of existing research in terms of intervention settings, artistic modalities, and targeted populations, a scoping review is considered the most appropriate methodology to map this complex evidence system. This study systematically maps the literature regarding arts-based interventions for female survivors of DV and IPV. Drawing on the Social Ecological Model (SEM)—conceptually informed by Bronfenbrenner's (16) ecological systems theory and operationalized for health promotion contexts by McLeroy et al. (17)—as an organizing framework, the review examines how these interventions operate across Individual, Interpersonal, and Community levels, and further explores the ecological alignment between the interventions’ stated objectives and the quantitative evaluation metrics employed. Ultimately, this research aims to provide methodological insights to inform the future development of a more ecologically sensitive and survivor-centered public health evaluation framework for arts interventions, thereby facilitating their integration into scalable, cross-sector support systems.

## 2 Methods

### 2.1 Study design

This scoping review was conducted in accordance with the methodological framework established by the Joanna Briggs Institute (JBI) for scoping reviews (18). To ensure transparency and reproducibility, the reporting process strictly adhered to the Preferred Reporting Items for Systematic reviews and Meta-Analyses extension for Scoping Reviews (PRISMA-ScR) guidelines (19). All methodological decisions, including the search strategy, eligibility criteria, and charting approach, were defined *a priori*. As this study involved only publicly available data from previously published literature, institutional ethics approval was not required.

### 2.2 Information sources and search strategy

A comprehensive systematic search was carried out across four electronic databases: Web of Science Core Collection (WoS), Scopus, PubMed, and PsycINFO. The search encompassed all relevant literature published up to May 27, 2025. Boolean operators were utilized to combine terms related to domestic violence (e.g., “intimate partner violence,” “victim of abuse”) and arts-based interventions (e.g., “art therapy,” “creative expression”), with syntax tailored to the specific indexing rules of each database. The search was restricted to original peer-reviewed articles published in English. Full search strings are provided in Supplementary material 1. Additionally, forward and backward citation tracking was conducted for all included articles to ensure comprehensive coverage.

### 2.3 Eligibility criteria

Eligibility criteria were developed using the Population, Concept, and Context (PCC) framework specific to scoping reviews (18). The complete criteria for inclusion and exclusion are presented in Table 1. Studies were included if they met the following criteria: (1) Population: Adult female survivors affected by domestic or intimate partner violence; (2) Concept: Interventions in which art, creative expression, or participatory arts served as a core therapeutic or psychosocial component, explicitly excluding interventions in which verbal psychotherapy constituted the sole or primary modality without a substantive arts-based component; and (3) Context: No restrictions were placed on the intervention setting (e.g., clinical, community, NGO, or digital spaces) to capture the diverse ecological implementation of these practices. Furthermore, eligible studies had to be original empirical research employing qualitative, quantitative, or mixed-methods designs. Non-empirical publications, such as reviews, commentaries, conference abstracts, and grey literature, were excluded from this scoping review.

**Table 1:** Inclusion and exclusion criteria based on the PCC framework.

PCC domain	Inclusion criteria	Exclusion criteria
Population (P)	Adult female survivors affected by DV or IPV.	Studies focusing exclusively on minors, male perpetrators/victims, or populations where DV/IPV status is unspecified.
Concept (C)	Interventions utilizing arts-based or creative modalities (e.g., visual arts, music, dance) as the primary psychosocial, recovery-oriented, or therapeutic tool.	Interventions unrelated to arts/creativity, or where art is merely a peripheral activity rather than the core mechanism.
Context (C)	Any service delivery setting (e.g., community NGOs, shelters, clinical outpatient, telehealth) across any geographical location.	N/A
Types of Evidence	Original empirical research, including qualitative, quantitative, and mixed-methods designs.	Non-empirical publications (e.g., review articles, editorials, book chapters, conference abstracts).
Language	Published in peer-reviewed journals and written in English.	Non-English publications or grey literature.

PCC, Population, Concept, Context. The inclusion criteria were designed to capture the broad spectrum of arts-based interventions, with a specific focus on those fostering psychosocial recovery and community reintegration, alongside clinical symptom-oriented approaches. N/A, Not applicable.

### 2.4 Study selection

**Figure 1:**
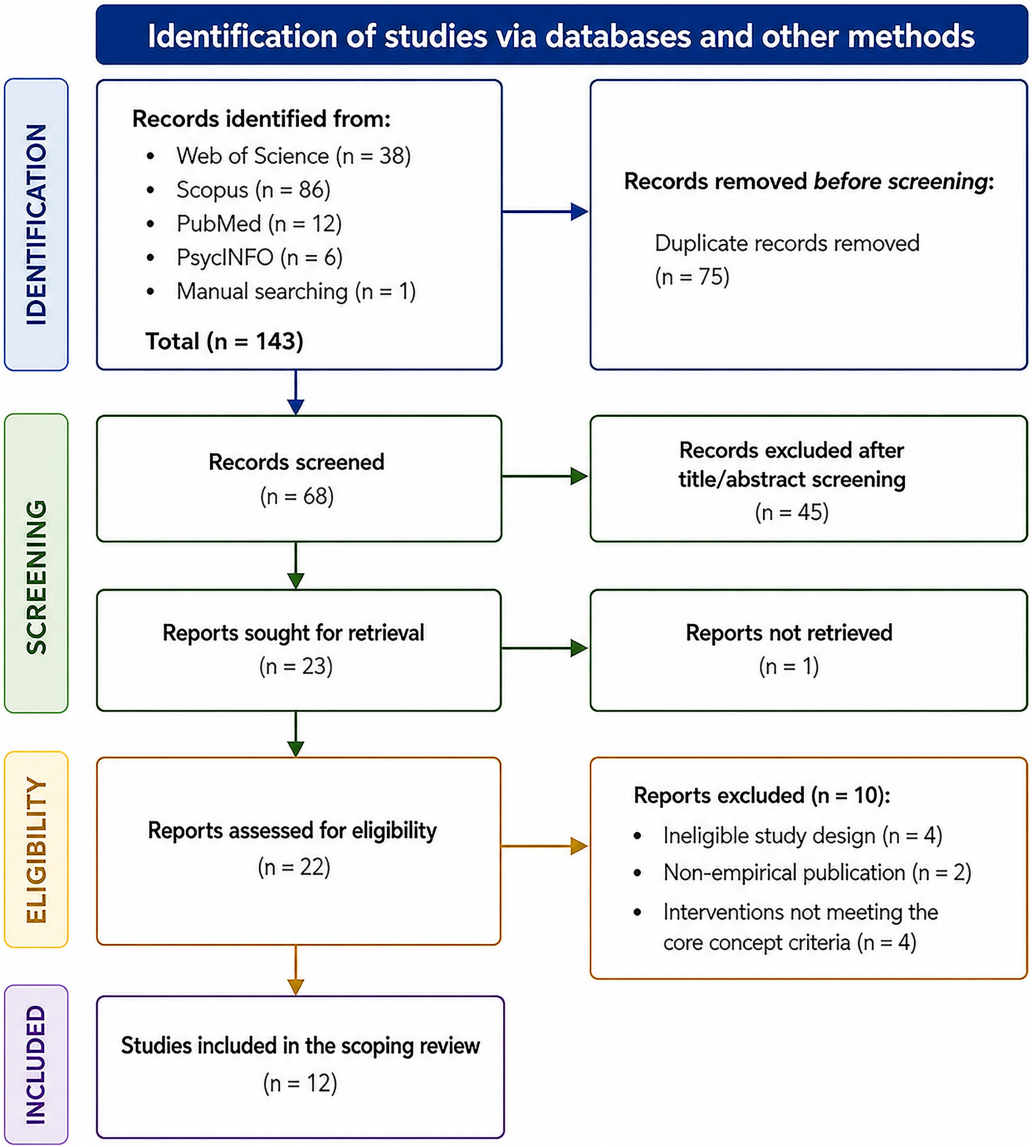
PRISMA-ScR flow diagram of the study selection process.

All retrieved records were imported into EndNote 20, and duplicates were removed. Two independent reviewers conducted a two-stage screening process. Initially, titles and abstracts were assessed against the established PCC criteria to determine relevance. This was followed by a full-text evaluation of potentially eligible articles to confirm final inclusion. Any discrepancies between the reviewers at either stage were resolved through iterative discussion until consensus was reached. The complete study selection process is summarized in the PRISMA-ScR flow diagram (Figure 1). Consistent with the primary objective of a scoping review—which is to map the extent and nature of available evidence rather than to evaluate intervention efficacy—a formal methodological quality appraisal of the included studies was not conducted (20).

### 2.5 Data charting process

Two reviewers independently charted the data using a standardized extraction form developed iteratively by the research team. Beyond standard bibliometric information and participant characteristics, data charting focused on two analytical dimensions: (1) primary ecological focus and (2) ecological alignment between intervention objectives and quantitative evaluation metrics. Discrepancies arising during the charting process were resolved through discussion until consensus was achieved.

**Figure 2:**
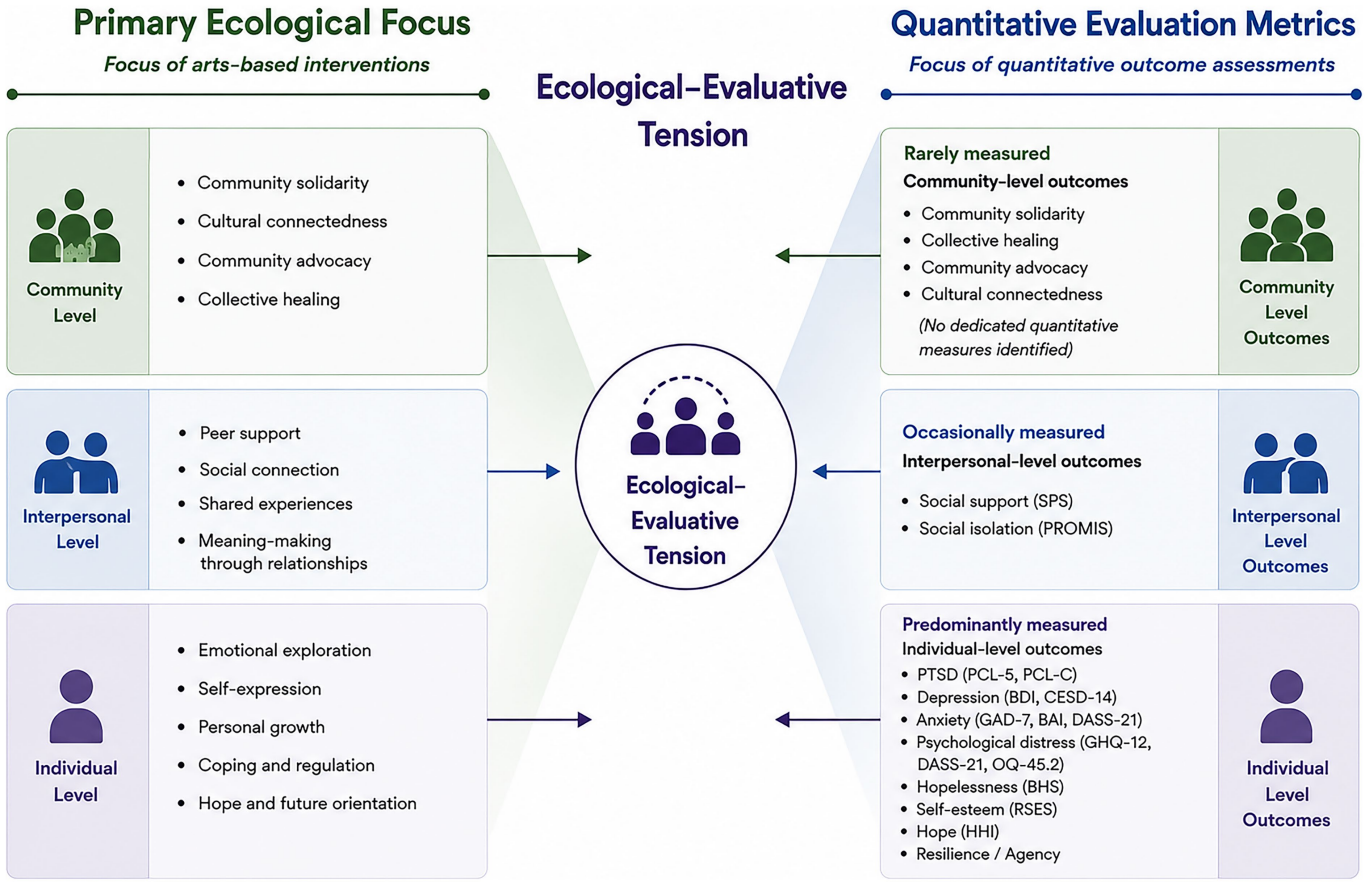
Conceptual illustration of potential relationships between intervention ecology and outcome evaluation.

Ecological classifications and alignment categories were developed inductively through an iterative review of the included studies, drawing strictly on the principles of the Social Ecological Model (17). Classification decisions were informed by the primary intervention objectives explicitly stated by the original study authors, alongside reported outcomes and implementation contexts. When multiple ecological levels were targeted, classification was based on the dominant or most explicitly stated intervention objective identified by the study authors. Where uncertainty arose, particularly in studies spanning multiple ecological levels or employing outcome measures that only partially reflected the stated intervention objectives, classifications were discussed and refined through reviewer consensus to enhance transparency and consistency in the evidence-mapping process (Figure 2).

Ecological alignment was defined as the degree of correspondence between a study’s primary ecological focus and the quantitative outcome measures used for evaluation. Four alignment categories were inductively developed through iterative review of the included studies. Specifically, (1) Congruent referred to studies in which the intervention objectives and quantitative outcome measures operated at the same ecological level; (2) Partially Congruent referred to studies in which interventions targeted multiple ecological domains and the quantitative measures captured at least one, but not all, of the intended dimensions; (3) Evaluative Mismatch referred to studies in which the primary intervention focus was situated at the interpersonal or community level, yet evaluation relied exclusively on individual-level psychological or clinical outcome measures. To minimize classification bias, studies were not categorized as Partially Congruent when quantitative outcomes reflected only downstream individual symptoms while the intervention’s primary objectives explicitly emphasized interpersonal, relational, or collective processes that were not directly operationalized within the evaluation framework; such cases were classified as Evaluative Mismatch. Finally, (4) Qualitative Evaluation referred to studies that employed exclusively qualitative methods and did not report formal quantitative outcome measures. It is important to note that these alignment categorizations are intended solely as descriptive analytical tools for mapping methodological patterns and operational dynamics across the literature, rather than as indicators of methodological quality or intervention efficacy. In addition, commonly reported findings were descriptively summarized across ecological levels to complement the evidence mapping.

This scoping review was motivated by the observation that evaluations of community arts interventions frequently rely on individual-level psychological outcomes, even when interventions explicitly target interpersonal relationships and community processes. The authors approached the review from an interdisciplinary perspective informed by art practice, public health, and gender studies, which may have shaped the interpretation of evidence. To enhance analytical rigor and minimize interpretive bias, all ecological classifications and coding decisions were independently reviewed by two reviewers and finalized through consensus, following the procedures described above.

### 2.6 Evidence mapping and analytical strategy

Due to the substantial heterogeneity in study designs, intervention settings, and outcome measures across the included literature, a quantitative meta-analysis was deemed unfeasible. Accordingly, an evidence mapping approach was adopted as an analytical strategy within the broader scoping review framework to systematically characterize the distribution, ecological orientation, and methodological features of the included studies (21). Data were iteratively synthesized to map the operational contexts of arts-based interventions alongside their evaluative frameworks.

During synthesis, intervention aims and reported outcomes were inductively grouped into Individual, Interpersonal, and Community levels to facilitate descriptive ecological mapping across studies. The synthesis specifically explored the alignment between the ecological focus of the interventions and the quantitative metrics employed for outcome assessment, culminating in a critical examination of prevailing methodological orientations in the field. The analytical synthesis remained descriptive in nature and was intended to map patterns across studies rather than to infer intervention effectiveness or causal mechanisms.

## 3 Results

### 3.1 Study selection

The initial systematic search across the four electronic databases, supplemented by manual searching, yielded a total of 143 records. Following the removal of 75 duplicates, 68 unique records were screened by title and abstract, resulting in the exclusion of 45 articles that did not meet the preliminary inclusion criteria. The remaining 23 reports were sought for retrieval, of which one could not be obtained. Full-text assessment of the remaining 22 articles was conducted against the predefined Population, Concept, and Context (PCC) criteria. During this phase, 10 articles were excluded due to ineligible study design (*n* = 4), non-empirical publication type (*n* = 2), and interventions not meeting the core concept criteria (*n* = 4). Ultimately, 12 empirical studies were included in the final scoping review. The complete study selection process is illustrated in the PRISMA-ScR flow diagram (Figure 1).

### 3.2 Characteristics of included studies

The 12 included studies spanned two decades, with publication years ranging from 2005 to 2025. Earlier publications were relatively infrequent, comprising two studies (16.7%) published between 2005 and 2010 and one study (8.3%) published between 2011 and 2015. In contrast, four studies (33.3%) were published between 2016 and 2020, and five studies (41.7%) between 2021 and 2025, indicating an increasing concentration of publications in recent years. Geographically, half of the studies (*n* = 6, 50.0%) originated from the United States, followed by Canada (*n* = 2, 16.7%). The remaining studies (*n* = 4, 33.3%) were distributed across Ecuador, India, Turkey, and the United Kingdom.

Regarding research methodology, mixed-methods designs were the most frequently employed approach (*n* = 7, 58.3%), followed by qualitative designs (*n* = 3, 25.0%) and quantitative designs (*n* = 2, 16.7%). Sample sizes across the studies varied considerably, with six studies (50.0%) reporting sample sizes of fewer than 10 participants, and four studies (33.3%) reporting samples between 10 and 49 participants. Two studies (16.7%) involved 100 or more participants, reflecting the predominantly small-scale and exploratory nature of this evidence base.

In terms of intervention delivery, most arts-based interventions were delivered within community-integrated settings, including domestic violence shelters, transitional housing facilities, community centers, NGO-supported programs, and telehealth-based support platforms (*n* = 9, 75.0%). Two studies (16.7%) employed individual arts-based therapeutic approaches, while one study (8.3%) was conducted within a clinical healthcare setting. Interventions were predominantly delivered in group-based formats (*n* = 9, 75.0%), with two studies (16.7%) employing individual therapy formats and one study (8.3%) using a virtual group delivery model (Table 2). The foundational characteristics and empirical profiles of all 12 included studies are summarized in Table 3.

**Table 2:** Descriptive characteristics of included studies (*N* = 12).

Study characteristics	Count (n)	Percentage (%)
Publication period		
2005–2010	2	16.7
2011–2015	1	8.3
2016–2020	4	33.3
2021–2025	5	41.7
Study design		
Mixed-methods	7	58.3
Qualitative	3	25.0
Quantitative	2	16.7
Country of origin		
United States	6	50.0
Canada	2	16.7
Ecuador	1	8.3
India	1	8.3
Turkey	1	8.3
United Kingdom	1	8.3
Sample size		
N < 10	6	50.0
10 ≤ N < 50	4	33.3
N ≥ 100	2	16.7
Intervention delivery format		
Group-based	9	75.0
Individual-based	2	16.7
Virtual group-based	1	8.3

Percentages may not sum to exactly 100% due to rounding. Studies were categorized according to publication period, methodological design, country of origin, sample size, and primary intervention delivery format.

**Table 3:** Study characteristics and key findings of included studies.

Study (Year), country	Design & sample	Participant population	Arts modality & delivery format	Duration	Key reported outcomes
Allen (29), USA	Mixed-methods (*N* = 11)	Marginalized women survivors of domestic violence	Multimodal arts-based healing program (group)	8 weeks	Reduced PTSD symptoms and facilitated collective witnessing, emotional expression, and identity reconstruction
Bird (31), UK	Qualitative (*N* = 8)	Women survivors of domestic abuse (24–53 years)	Collage and reflective arts practice (group)	12 weeks	Supported trauma processing through sensory and visual expression
Cherian (26), India	Mixed-methods (*N* = 135)	Dalit and minority women residing in shelters (Mean age = 28.4)	Facilitative visual arts intervention (group)	6 weeks	Reduced symptoms of anxiety and depression
Frohmann (22), USA	Qualitative (*N* = 24)	Immigrant women survivors of domestic violence	Photovoice intervention (group)	6 months	Enhanced safety meaning-making and community advocacy
Giesbrecht (23), Canada	Mixed-methods (*N* = 19)	Indigenous women survivors of domestic violence	Indigenous cultural-arts healing program (group)	10 weeks	Enhanced resilience, agency, cultural connectedness, and social support
Ikonomopoulos (25), USA	Quantitative single-case design (*N* = 3)	Hispanic women survivors of domestic violence (Mean age = 30)	Creative journal arts therapy (individual)	6–9 sessions	Reduced psychological distress and hopelessness
Aktaş Özkafacı (24), Turkey	Mixed-methods (*N* = 8)	Women diagnosed with PTSD following domestic violence (39–45 years)	Marbling art psychotherapy (group)	14 weeks	Reduced depression, anxiety, and hopelessness symptoms
Özümerzifon (32), USA	Mixed-methods RCT (*N* = 45; intervention = 25, control = 20)	Black and Latina survivors of intimate partner violence (23–48 years)	Dance/movement program (virtual group)	12 sessions over 6 weeks	Reduced social isolation and strengthened virtual peer connection
Sabina (28), Ecuador	Mixed-methods (*N* = 345)	Latina survivors of intimate partner violence	Community-based music and therapeutic support program (group)	Ongoing	Improved psychological well-being and strengthened community solidarity
Skop (33), Canada	Qualitative (*N* = 6)	Women accessing domestic violence prevention services (34–65 years)	Visual arts therapy using watercolor, charcoal, and clay (group)	12 weeks	Promoted empowerment and survivor identity reconstruction
Teague (27), USA	Mixed-methods (*N* = 7)	Women survivors of domestic violence (19–45 years)	Music therapy and clay-based intervention (group)	10 weeks	Improved mood and strengthened peer support networks
Vela (30), USA	Quantitative single-case design (*N* = 3)	Hispanic women survivors of intimate partner violence (Mean age = 27)	Creative journal arts therapy (individual)	9 sessions	Improved self-esteem and hope

N represents the total study sample. RCT, Randomized Controlled Trial. PTSD, Post-Traumatic Stress Disorder. Studies are presented chronologically and include qualitative, quantitative, and mixed-methods evidence examining arts-based interventions for women survivors of domestic violence or intimate partner violence.

### 3.3 Ecological contexts and delivery of arts-based interventions

The included studies described arts-based interventions involving populations experiencing diverse social and structural vulnerabilities. Target groups included women in shelters, Indigenous women, Latina and Hispanic survivors, recent immigrants, and women managing the isolation associated with the COVID-19 pandemic.

The interventions incorporated a wide range of creative modalities, including painting, clay work, collage, traditional marbling (Ebru), photovoice, dance and movement practices, music therapy, Indigenous beading, traditional medicines, and healing rituals. For example, photovoice approaches were frequently utilized within advocacy-oriented community contexts (22), whereas culturally grounded healing practices such as Indigenous beading and medicines were reported in studies involving Indigenous women (23).

Authors described intervention goals operating across multiple domains of participants’ experiences. At the individual level, interventions were often described as focusing on emotional exploration, self-expression, and distress tolerance [e.g., (24, 25)]. At the interpersonal level, studies frequently reported aims related to establishing safe peer environments, fostering relational interaction, and building social support networks within transitional or shelter settings [e.g., (26, 27)]. At the community level, authors described intervention aims related to collective healing, public advocacy, and cultural reconnection within marginalized communities [e.g., (22, 23)]. Table 4 outlines the equity-positioned populations, intervention settings, and primary ecological focus described within each study.

**Table 4:** Reported intervention focuses, delivery contexts, and ecological levels of included studies.

Study (Year)	Equity-positioned population	Structural context & setting	Reported intervention focus	Ecological level
Giesbrecht (23)	Indigenous women	Indigenous healing environment	Cultural connectedness, identity reclamation, and intergenerational healing	Community
Frohmann (22)	Immigrant women	Public advocacy spaces	Safety meaning-making and community advocacy	Community
Sabina et al. (28)	Latina survivors	NGO and community support networks	Collective resilience and community solidarity	Interpersonal
Allen and Wozniak (29)	Marginalized women survivors	Community agency	Collective healing and group connectedness	Interpersonal
Bird (31)	Women survivors of domestic abuse	Voluntary sector support service	Shared narrative expression and peer transition	Interpersonal
Skop (33)	Women utilizing domestic violence prevention services	Prevention agency and community setting	Identity reconstruction and mutual support	Interpersonal
Cherian and Reshmy (26)	Dalit and minority women	Institutional shelters	Safe peer environments and supportive social interaction	Interpersonal
Teague (27)	Women survivors, including recent immigrants	Transitional housing program	Peer support and interpersonal connection *(Note: Outcome focused on individual mood)*	Individual
Özümerzifon (32)	Black and Latina survivors	Telehealth and digital platforms	Virtual peer connection and reduction of social isolation *(Note: Outcome focused on individual distress)*	Individual
Ikonomopoulos (25)	Hispanic women survivors	Domestic violence shelter	Distress tolerance and emotional coping	Individual
Aktaş Özkafacı and Eren (24)	Women diagnosed with PTSD following domestic violence	Psychiatric clinic	Trauma processing and symptom reduction	Individual
Vela (30)	Hispanic women survivors	Outpatient counseling clinic	Hope enhancement and self-esteem restoration	Individual

Ecological levels were inductively classified based on the reported primary focus and operational dynamics of each intervention, drawing on principles from the Social Ecological Model (17). Classification consensus was reached through discussion between two reviewers. Studies are ordered by Ecological Level to facilitate descriptive mapping across Community, Interpersonal, and Individual domains.

### 3.4 Mapping of intervention domains and outcome evaluation approaches

The review descriptively mapped the qualitative themes reported in the included studies alongside the quantitative outcome measures utilized to evaluate arts-based interventions. Qualitative findings across the studies frequently reported themes related to peer support, community solidarity, identity reconstruction, and collective healing. As summarized in Table 5, these themes spanned individual, interpersonal, and community ecological levels, with interpersonal and community-focused dimensions consistently emerging across diverse intervention contexts. However, as demonstrated by the alignment analysis in Table 6, these broader ecological dimensions were rarely captured through dedicated quantitative outcome measures, indicating an evaluative tension between intervention objectives and quantitative outcome measures.

**Table 5:** Summary of commonly reported themes across ecological levels in the included studies.

Ecological level	Commonly reported themes	Illustrative findings	Representative studies
Individual	Emotional Expression & Trauma Processing	Non-verbal externalization of trauma; sensory engagement with art materials; emotional coping through creative expression	(24, 31, 32)
	Identity Reconstruction	Rebuilding self-worth and survivor identity; reclaiming personal agency; self-forgiveness	(29, 31, 33)
	Hope & Future Orientation	Positive future orientation; goal-setting capacity; reduced hopelessness	(24, 28, 29)
Interpersonal	Peer Support & Mutual Witnessing	Safe group environment; reduced social isolation; shared understanding among survivors	(27, 29, 32)
	Shared Narrative Expression	Co-creation of meaning through artistic media; moving from private to shared experience	(26, 31, 33)
	Mutual Aid & Sisterhood	Reciprocal support and solidarity; sustained peer connectedness beyond intervention sessions	(26, 28)
Community	Cultural Connectedness & Intergenerational Healing	Reclamation of Indigenous identity; traditional healing practices; intergenerational trauma addressed through cultural arts	(23)
	Community Solidarity & Collective Advocacy	Collective resilience; shared meaning-making around safety; public advocacy through participatory arts	(22, 28)

Themes summarize commonly reported findings identified during data charting and are presented descriptively to complement the ecological mapping (Table 4) and alignment analysis (Table 6). Studies are grouped according to the Social Ecological Model (17).

**Table 6:** Alignment between intervention targets and outcome evaluation approaches across included studies.

Study (Year)	Study design	Quantitative evaluation metrics	Ecological focus of intervention objectives	Alignment between intervention objectives and quantitative evaluation metrics
Ikonomopoulos (25)	Quantitative	OQ-45.2 (Psychological Functioning); BHS (Hopelessness); Brief Resilience Scale (Resilience)	Individual	**Congruent** (Psychological functioning, hopelessness, and resilience outcomes were directly aligned with the intervention’s emotional coping and recovery objectives.)
Aktaş Özkafacı and Eren (24)	Mixed-methods	BDI (Depression); BAI (Anxiety); BHS (Hopelessness)	Individual	**Congruent** (Depression, anxiety, and hopelessness outcomes were directly aligned with the intervention’s trauma-processing and symptom-reduction objectives.)
Özümerzifon (32)	Mixed-methods	PROMIS (Anxiety, Depression, Social Isolation); HRV (Physiological Regulation)	Individual	**Congruent** (Psychological and physiological outcomes were directly aligned with the intervention’s mental health and emotional regulation objectives.)
Teague (27)	Mixed-methods	VAS (Mood)	Individual	**Congruent** (Mood outcomes were directly aligned with the intervention’s objective of reducing emotional distress and improving well-being.)
Vela (30)	Quantitative	RSES (Self-Esteem); HHI (Hope)	Individual	**Congruent** (Self-esteem and hope outcomes were directly aligned with the intervention’s empowerment and recovery objectives.)
Cherian and Reshmy (26)	Mixed-methods	DASS-21 (Depression, Anxiety, Stress)	Interpersonal	**Partially Congruent** (Individual psychological distress was assessed, although broader interpersonal and social well-being objectives were not directly measured.)
Giesbrecht (23)	Mixed-methods	PCL-5 (PTSD Symptoms); CESD-14 (Depressive Symptoms); GAD-7 (Anxiety Symptoms); Resilience Measures; Agency Measures	Community	**Partially Congruent** (Resilience and agency outcomes partially reflected community-oriented objectives, although broader collective and community-level changes were not directly assessed.)
Sabina et al. (28)	Mixed-methods	GHQ-12 (Psychological Distress); SPS (Social Support)	Interpersonal	**Partially Congruent** (Interpersonal social support was assessed, although broader relational recovery and collective resilience processes were not fully captured.)
Allen and Wozniak (29)	Mixed-methods	PCL-C (PTSD Symptoms)	Interpersonal	**Evaluative Mismatch** (Individual PTSD symptoms were used exclusively to evaluate interpersonal group bonding and collective healing processes, resulting in a mismatch between intervention objectives and outcome measurement.)
Bird (31)	Qualitative	N/A	Interpersonal	**Qualitative Evaluation** (No quantitative outcome measures were reported.)
Frohmann (22)	Qualitative	N/A	Community	**Qualitative Evaluation** (No quantitative outcome measures were reported.)
Skop (33)	Qualitative	N/A	Interpersonal	**Qualitative Evaluation** (No quantitative outcome measures were reported.)

Studies are grouped according to methodological alignment category. Alignment reflects the degree of correspondence between reported intervention foci and quantitative outcome metrics, inductively assessed by the review team where both qualitative and quantitative components were available. Qualitative-only studies did not employ formal quantitative outcome measures. N/A, Not applicable. BAI, Beck Anxiety Inventory (34); BDI, Beck Depression Inventory (35); BHS, Beck Hopelessness Scale (36); CESD-14, Center for Epidemiologic Studies Depression Scale–14 (37, 38); DASS-21, Depression Anxiety Stress Scales–21 (39); GAD-7, Generalized Anxiety Disorder–7 (40); GHQ-12, General Health Questionnaire (41); HHI, Herth Hope Index (42); OQ-45.2, Outcome Questionnaire (43); PCL-C, PTSD Checklist–Civilian Version (44); PCL-5, PTSD Checklist for DSM-5 (45); PROMIS, Patient-Reported Outcomes Measurement Information System (46); RSES, Rosenberg Self-Esteem Scale (47); SPS, Social Provisions Scale (48); VAS, Visual Analogue Scale (49).

Mapping the authors’ explicitly stated intervention objectives against their quantitative evaluation metrics identified a spectrum of ecological alignment across the included studies. Five studies demonstrated Congruent alignment, wherein quantitative metrics directly matched the individual-level focus of the interventions, effectively capturing targeted clinical symptoms such as PTSD, depression, anxiety, or compromised self-esteem [e.g., (25, 27)].

However, as interventions expanded to target interpersonal and community-level objectives, evaluative tensions emerged. Three mixed-methods studies demonstrated Partially Congruent alignment. In these cases, interventions targeted broader social well-being or collective resilience, but quantitative tools only captured a fraction of these domains (e.g., social support or individual stress), leaving other collective objectives unmeasured [e.g., (26, 28)]. Furthermore, one study represented an Evaluative Mismatch, wherein the intervention was explicitly designed around interpersonal collective healing, yet the quantitative evaluation relied exclusively on individual-level clinical symptom metrics (29). The detailed ecological alignment between intervention targets and quantitative outcome measures for each study is mapped in Table 6. These patterns suggest a methodological tendency whereby intervention processes frequently extend across broader ecological levels, whereas quantitative outcome assessments remain disproportionately anchored to individual-level clinical indicators.

## 4 Discussion

### 4.1 Arts-based interventions extend beyond individual recovery

This scoping review mapped the landscape of arts-based interventions for women survivors of domestic violence and identified substantial diversity in both intervention contexts and intended outcomes. Consistent with contemporary public health perspectives, the included studies demonstrated that arts-based interventions were rarely confined to symptom management alone. Instead, many programs were implemented within community agencies, shelters, advocacy organizations, and culturally specific support environments, reflecting broader goals related to social participation, identity reconstruction, peer connection, and community engagement.

Mapping intervention foci through the Social Ecological Model revealed that arts-based interventions operated across multiple ecological levels. While several interventions focused primarily on individual recovery processes such as emotional coping, trauma processing, and self-esteem restoration, a larger proportion emphasized interpersonal and community-oriented objectives, including peer support, collective healing, cultural connectedness, mutual aid, and community solidarity. These findings support previous arguments that recovery from domestic violence extends beyond the reduction of psychological symptoms and frequently involves rebuilding social relationships, restoring agency, and re-establishing connections with supportive communities.

The ecological distribution observed in this review also highlights the distinctive role of arts-based approaches within public health practice. Unlike many clinically oriented interventions that primarily target intrapersonal symptom reduction, arts-based programs often function as socially embedded practices that facilitate both personal expression and relational engagement. This suggests that their potential contribution lies not only in improving psychological wellbeing but also in creating opportunities for social participation and collective recovery, thereby serving as a crucial component of coordinated, cross-sector responses bridging health and social systems.

### 4.2 Patterns of alignment between intervention focus and outcome evaluation

Beyond mapping intervention ecology, this review critically examined the ecological alignment between stated intervention objectives and quantitative evaluation metrics. The findings revealed that the degree of alignment varied systematically depending on the ecological ambition of the intervention.

Studies focusing primarily on individual trauma processing, symptom reduction, or emotional coping demonstrated strong Congruence between intervention goals and evaluation metrics [e.g., (24, 30)]. When the objective was to alleviate individual psychological distress, standardized clinical measures performed effectively and appropriately.

However, the transition to interpersonal and community-level interventions revealed notable methodological tension. Several studies achieved Partial Congruence by incorporating measures like social support (28) or resilience (23). Yet, even in these studies, clinical symptom measures (e.g., depression, anxiety) remained disproportionately prominent despite the authors’ explicitly stated goals of fostering broader social well-being or community solidarity. For example, Cherian and Reshmy (26) established psychological recovery and social well-being as central intervention objectives, yet quantitative evaluation relied primarily on the DASS-21, leaving the interpersonal domains of their objectives quantitatively unassessed.

The most striking evidence of this tension—Evaluative Mismatch—occurred when relational or collective objectives were evaluated exclusively through individual-level clinical tools. A particularly illustrative example is provided by Allen and Wozniak (29). While their intervention was explicitly conceptualized as a “social, spiritual, and cultural process” designed to foster “group cohesiveness” and collective healing, the quantitative evaluation relied exclusively on the PCL-C, providing limited direct assessment of the intended collective processes. This may reflect a broader tendency to rely on established clinical outcome measures even when intervention objectives extend beyond the individual level.

Qualitative-only studies [e.g., (22, 31)] represented a different methodological strategy. By foregoing standardized outcome measurement, these studies explored participants’ experiences of empowerment, community advocacy, and social connection, further highlighting the domains currently underrepresented in quantitative evaluative frameworks.

### 4.3 Understanding the ecological–evaluative tension

Viewed through the Social Ecological Model, this tension may reflect a potential misalignment between the ecological level at which interventions operate and the ecological level at which outcomes are evaluated. Crucially, when researchers explicitly identify interpersonal or community-level processes as primary intended domains of change, the absence of corresponding quantitative indicators limits the extent to which these stated objectives can be empirically verified or systematically compared across studies. This represents a methodological concern independent of whether community-level outcomes are ultimately more or less important than individual-level psychological change. Community-focused interventions often aimed to strengthen cultural connectedness, collective healing, social participation, or peer solidarity, yet these outcomes were rarely assessed through dedicated quantitative measures. Similarly, interpersonal outcomes such as social support and relational connection appeared less frequently in evaluation frameworks than individual-level psychological indicators.

Importantly, this interpretation should be approached cautiously. The current evidence base does not establish that existing evaluation approaches are inherently inadequate; clinical measures of depression, anxiety, trauma symptoms, and distress remain highly relevant, particularly when interventions explicitly target psychological recovery. Furthermore, some relational constructs were already represented through measures of social support, social isolation, self-esteem, and hope. Nevertheless, the consistency with which qualitative findings emphasized peer connection, mutual support, cultural belonging, and collective recovery across diverse contexts suggests that existing quantitative evaluation frameworks may not fully capture the breadth of changes reported by participants. However, it must also be acknowledged that qualitative findings are inherently susceptible to social desirability and researcher interpretation biases, highlighting the need for a more balanced, multi-level evaluation strategy.

### 4.4 Implications for evaluation and public health practice

These findings have important implications for public health evaluation and policy development. For women survivors of domestic violence, recovery often involves navigating not only psychological distress but also social isolation, disrupted relationships, reduced autonomy, and broader structural vulnerabilities. Consequently, evaluation frameworks that focus predominantly on symptom reduction may underestimate the wider contributions of community-based and arts-based programs. Crucially, the inability to quantitatively capture these relational and community-level benefits may hinder the translation of innovative arts-based models into sustainable public health policy, limiting their integration into broader, cross-sector support systems.

Future research may benefit from expanding evaluation frameworks beyond individual-level symptom indicators while retaining the strengths of established clinical measures. This could include greater use of instruments assessing social support, social connectedness, community participation, collective efficacy, and survivor-defined indicators of recovery. Examples may include validated instruments such as the Social Provisions Scale (SPS) for assessing perceived social support and PROMIS measures of social isolation, alongside participatory approaches that capture survivor-defined recovery outcomes. Participatory evaluation approaches developed in collaboration with survivors may also provide valuable insight into outcomes that are meaningful within specific cultural and community contexts.

Rather than replacing clinical assessment, a more ecologically responsive evaluation framework would integrate psychological, relational, and community-level indicators. Such an approach may provide a more comprehensive understanding of how arts-based interventions contribute to recovery and wellbeing among survivors of domestic violence.

### 4.5 Limitations and future directions

Several limitations of this scoping review should be acknowledged. First, the review was restricted to peer-reviewed publications written in English, potentially excluding relevant evidence reported in grey literature or other languages. Second, substantial methodological heterogeneity across studies limited direct comparison between intervention approaches and outcome measures. Third, many included studies involved small sample sizes, with approximately half including fewer than 10 participants, reflecting the exploratory nature of the field and limiting the generalizability of findings. Furthermore, while this review identifies an evaluative mismatch wherein community or interpersonal outcomes frequently remain unmeasured, the current scoping methodology does not allow for causal inferences regarding its origins. Whether this tension reflects a lack of methodological awareness, practical resource constraints, or a scarcity of validated ecological measurement tools remains empirically unclear and warrants dedicated investigation in future research. Additionally, the final inclusion of only 12 eligible empirical studies highlights a significant limitation in the current evidence base. However, rather than undermining the review’s validity, this scarcity reflects the nascent stage of empirical research at the intersection of arts-based interventions and domestic violence recovery, thereby underscoring the critical necessity and timeliness of this scoping review in mapping the existing baseline for this emerging cross-sector field.

It is also worth noting that the COVID-19 pandemic represents a significant contextual factor for this field. Evidence consistently documented sharp increases in DV and IPV incidence during pandemic-related lockdowns (4), while simultaneously accelerating the shift toward virtual arts-based delivery modalities, as exemplified by Özümerzifon et al. (32). Future research may benefit from specifically examining how pandemic conditions shaped both the delivery and evaluation of arts-based interventions for survivors.

Additionally, the studies identified through this review were predominantly implemented in community or NGO settings, which may have systematically amplified the observed ecological–evaluative tension. Finally, the category of arts-based interventions encompasses a wide range of creative modalities, delivery settings, and participant populations. Consequently, the findings presented here should be interpreted as evidence of broad methodological patterns rather than modality-specific effects. Despite these limitations, this review provides a systematic overview of how arts-based interventions for women survivors of domestic violence are currently implemented and evaluated, while highlighting the critical need for developing more ecologically responsive and survivor-centered approaches to quantitative outcome assessment.

## 5 Conclusion

This scoping review mapped the landscape of arts-based interventions for women survivors of domestic violence, identifying 12 empirical studies that collectively demonstrate the ecological breadth and methodological diversity characterizing this field. Organized through the Social Ecological Model, the findings reveal that arts-based interventions frequently operate across Individual, Interpersonal, and Community levels simultaneously, addressing not only psychological distress but also peer connection, cultural belonging, identity reconstruction, and collective healing.

A central finding of this review is the presence of an ecological–evaluative tension: while many interventions were designed to facilitate change across interpersonal and community domains, quantitative outcome evaluation remained predominantly concentrated at the individual level, with interpersonal and community-level outcomes rarely represented through dedicated quantitative measurement tools. This pattern does not indicate a failure of existing clinical measures, which retain clear relevance for interventions targeting psychological recovery. Rather, it suggests an opportunity to broaden evaluation frameworks so that they more fully reflect the multi-level processes through which arts-based programs support survivor recovery.

For public health practice and policy, these findings underscore the importance of developing ecologically responsive evaluation approaches that integrate psychological, relational, and community-level indicators alongside established clinical measures. Such frameworks would better capture the wider contributions of community-based arts programs and support more accurate assessments of their value within survivor-centered, cross-sector public health systems.

As empirical attention to this intersectional field continues to grow, future research would benefit from larger and more diverse samples, greater methodological consistency, and increased collaboration with survivors in the co-design of evaluation tools. These findings carry practical implications for public health practitioners, service providers, and policymakers. When evaluation frameworks remain restricted to individual psychological metrics, the broader relational and community-level contributions of arts-based programs risk being systematically underrepresented in evidence syntheses and funding decisions. Aligning outcome evaluation strategies with the ecological objectives of these interventions may therefore strengthen the evidence base needed to support equitable, scalable, and ecologically informed domestic violence recovery programs within cross-sector public health systems.

## Data Availability

The original contributions presented in the study are included in the article/Supplementary material, further inquiries can be directed to the corresponding author.
