# Supplementary material for: Mapping ecological targets and outcome evaluations in arts-based interventions for women survivors of domestic violence: a scoping review
Source: Front Public Health. 2026 Jul 17;14:1897444. doi: 10.3389/fpubh.2026.1897444 (PMC13424411; doi:10.3389/fpubh.2026.1897444)
Supplement: Supplementary file 1 [file Table_1.DOCX]

Supplementary Material

# Supplementary Table

**Table. Database Search Strategy**

| Database | Search string strategy Boolean operators |
| --- | --- |
| WoS | TS= (("domestic violence" OR "family violence" OR "intimate partner violence" OR "violence survivor" OR "victim of abuse") AND ("art therapy" OR "arts-based intervention" OR "art intervention" OR "creative arts" OR "art expression" OR "arts-based program" OR "creative expression" OR "participatory art")) AND LA=(English) AND DT=(Article) |
| Scopus | TITLE-ABS-KEY ("domestic violence" OR "family violence" OR "intimate partner violence" OR "violence survivor" OR "victim of abuse") AND TITLE-ABS-KEY ("art therapy" OR "arts-based intervention" OR "art intervention" OR "creative arts" OR "art expression" OR "arts-based program" OR "creative expression" OR "participatory art") AND (LIMIT-TO (LANGUAGE, "English")) AND (LIMIT-TO (DOCTYPE, "ar")) |
| PubMed | (("domestic violence"[Title/Abstract] OR "family violence"[Title/Abstract] OR "intimate partner violence"[Title/Abstract] OR "violence survivor"[Title/Abstract] OR "victim of abuse"[Title/Abstract]) AND ("art therapy"[Title/Abstract] OR "arts-based intervention"[Title/Abstract] OR "art intervention"[Title/Abstract] OR "creative arts"[Title/Abstract] OR "art expression"[Title/Abstract] OR "arts-based program"[Title/Abstract] OR "creative expression"[Title/Abstract] OR "participatory art"[Title/Abstract])) AND (English[lang]) AND ("Journal Article"[Publication Type]) |
| PsyINFO | AB= ("domestic violence" OR "family violence" OR "intimate partner violence" OR "violence survivor" OR "victim of abuse") AND AB=("art therapy" OR "arts-based intervention" OR "art intervention" OR "creative arts" OR "art expression" OR "arts-based program" OR "creative expression" OR "participatory art") AND LA=English AND PT="Journal Article" |
